# Design and Testing of a Novel Nested, Compliant, Constant-Force Mechanism with Millimeter-Scale Strokes

**DOI:** 10.3390/mi14020480

**Published:** 2023-02-18

**Authors:** Xuejiao Qin, Shuaishuai Lu, Pengbo Liu, Peng Yan

**Affiliations:** 1 School of Mechanical Engineering, Qilu University of Technology (Shandong Academy of Sciences), Jinan 250353, China; 2 Shandong Institute of Mechanical Design and Research, Jinan 250031, China; 3 Key Laboratory of High-Efficiency and Clean Mechanical Manufacture, Ministry of Education, School of Mechanical Engineering, Shandong University, Jinan 250061, China

**Keywords:** compliant mechanism, constant force, V-shaped beam, bi-stable beam

## Abstract

This paper presents a novel nested, compliant, constant-force mechanism (CFM) that generates millimeter-scale manipulation stroke. The nested structure is utilized to improve the overall compactness of the CFM. A combination strategy of positive and negative stiffness is induced to generate constant force with a millimeter-level range. In particular, bi-stable beams are used as the negative stiffness part, and V-shaped beams are selected as the positive stiffness part, and they are constructed into the nested structures. With this, a design concept of the CFM is first proposed. From this, an analytical model of the CFM was developed based on the pseudo-rigid body method (PRBM) and chain beam constraint model (CBCM), which was verified by conducting a simulation study with nonlinear finite-element analysis (FEA). Meanwhile, a parametric study was conducted to investigate the influence of the dominant design variable on the CFM performance. To demonstrate the performance of the CFM, a prototype was fabricated by wire cutting. The experimental results revealed that the proposed CFM owns a good constant-force property. This configuration of CFM provides new ideas for the design of millimeter-scale, constant-force, micro/nano, and hard-surface manipulation systems.

## 1. Introduction

### 1.1. Background

With the rapid development of precision engineering fields, micro/nano manipulation technology is gradually developing in the direction of miniaturization and integration [1,2,3,4]. The emergence of compliant mechanisms is successfully and widely used in the field of micro/nano manipulation. This also provides an opportunity for the development of compliant constant force mechanisms (CFMs).

Many engineering applications urgently require a constant operating force to satisfy operating on a specific object in order to prevent damage and destruction of the target object. Scholars have proposed various methods to control the operating force by using force sensors to monitor and control the force in real-time, but this method is complex and difficult [5,6,7,8,9]. In recent years, scholars have developed CFMs to achieve a constant-force output [10,11,12,13]. Studies have shown that this method does not require complex control devices, has a simpler structure, and is easily operatable. Wang et al. [14] equipped a constant-force bi-stable micromachine to protect a micro-device, which had the function of force regulation and overload protection. When an unknown force was applied to the device, the constant force bi-stable micro-mechanism could quickly return to other stable states to protect the device. Chen et al. [15] combined the negative stiffness characteristics of the bi-stable mechanism with the positive stiffness characteristics of the linear spring to design an adjustable CFM for passively adjusting the contact force of the robot end-effector. The mechanism could adjust the magnitude of the constant force to suit different working situations by adjusting the pre-stress of the prior spring, which successfully avoided the damage to the target object caused by the contact force in an unknown environment.

### 1.2. Motivation

Based on this background, scholars also made a lot of achievements in the design and application of CFM. In the design of CFM, Xu [16] developed a micro-positioning platform with a large stroke by using the characteristics of a bi-stable mechanism, which can output a certain constant force within 4.44 mm and is widely used in the tiny precision operation of a micro-positioning platform. Ding et al. [17] designed a bending-based constant force module combined with a Z-shaped beam and a bi-stable beam, and the parameters of this mechanism were optimized to obtain better constant force performance. A novel CFM based on the combination of positive and negative stiffness mechanisms was also proposed [18], using a folded beam combined with a bi-stable beam. This mechanism was also used in an industrial deburring operation. In order to reduce the force drive, Tian et al. [19] designed a CFM with structural holes by improving the bi-stable beam and also connecting it in parallel with the folded beam with structural holes to combine a novel CFM with low mass and stiffness. Tolman et al. [20] similarly designed a novel compliant CFM using the positive and negative stiffness principle and proposed an adjustable version of this mechanism was proposed. Zhang et al. [21] proposed a curved-beam-based quasi-constant-force mechanism with an appropriate configuration of circular arc elements to achieve a large range of quasi-constant-force output. Constant force mechanisms have also been applied in a variety of work situations, especially in the field of micro/nano manipulation. Liu and Xu [22,23,24] designed three compliant constant force micro-gripping devices using the positive and negative stiffness principle. Zhang et al. [25] designed a novel CFM-based compliant parallel gripper, which achieved constant-force gripping through the combination of a bi-stable beam and a flexible folding beam and had both active and passive constant-force characteristics in the X and Y directions. Ye et al. [26] designed a two-stage flexible, constant-force micro-gripper to achieve constant-force output through a combination of bi-stable and straight beams. Wang et al. [27] used a combination of positive and negative stiffness mechanisms to design a CFM-type precision positioning stage using a piezoelectric ceramic through a displacement amplifier, which could output a constant force for application in biological cell manipulation. Chen and Lan [28] designed an irregular constant-force slide buckle mechanism using the shape optimization method and verified the constant-force performance of the mechanism through experiments. Weight et al. [29] designed an electrically connected, constant-force device that maintains an optimal constant force with the aid of the contact surface of the cam and the geometry of the flexible segment. Meaders and Mattson [30] further optimized this structure to make its constant-force output characteristics more reasonable.

*Although there are many designs for CFM, existing CFMs have the limitations of a low output constant force, a short constant-force stroke, or an overall non-compact structure. Nowadays, many advanced applications require touching or machining hard material, such as grating ruling, robotic grinding, polishing, etc. Therefore, it is necessary to develop a CFM with a large output force and a constant-force stroke for this processing.*

### 1.3. Contribution

In this article, we designed a novel nested type of flexible CFM, which has the performance of outputting a large constant force within a millimeter-level stroke. This nested structure can well improve the overall compactness. In this design, we used the positive and negative stiffness principle, where a double V-shaped beam was selected as the positive stiffness mechanism, and a double bi-stable beam was the negative stiffness mechanism, connected in parallel to form a zero-stiffness mechanism.

The remainder of this paper is organized as follows: The design procedure of the compliant mechanism and the model analysis are introduced in Section 2. In Section 3, the finite element analysis and parametric sensitivity analysis of the CFM are demonstrated. In Section 4, the performance of the CFM is experimentally verified. Finally, this paper concludes with some concluding remarks in Section 5.

## 2. Design and Modeling Analysis of a Compliant CFM

In this article, we investigated a compliant CFM for millimeter-scale micro/nano operation, designed with the motivation of being able to output a large constant force in the millimeter range. It prevents damage and destruction to the target object due to force instability during operation and has the property of protecting the target object. Meanwhile, we hope that the design of this mechanism can meet the needs of practical applications.

### 2.1. Mechanism Design

**Figure 1:**
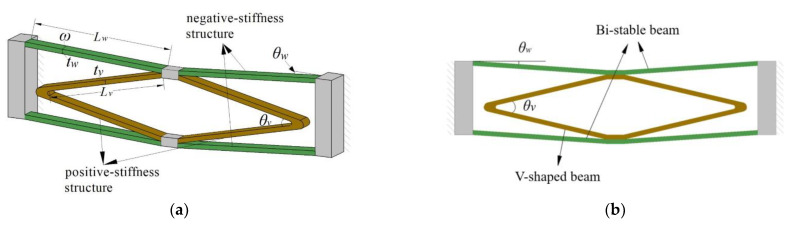
A schematic of the CFM: (**a**) axonometric view; (**b**) front view.

In the literature [22], a constant force-based gripper has been proposed, designed by combining a bi-stable beam and a flexible straight beam. This CFM is capable of producing a constant-force stroke of 220 µm. Based on this design, the flexible straight beam was changed to a double V-shaped beam, and a bi-stable beam was added on the other side, as shown in Figure 1, with the improved constant-force mechanism having a constant-force stroke of 1 mm.

The designed compliant CFM includes bi-stable and V-shaped beams, as shown in Figure 1a, and the whole mechanism consists of an internal double V-shaped beam and an external bi-stable beam, with the double V-shaped beam structure nested between the bi-stable beams. This design concept improves the overall stiffness and compactness of the mechanism. The bi-stable beam, as the part of CFM that generates negative stiffness, is a flexible straight beam with an initial angle. The buckling phenomenon occurs when the amount of deformation exceeds a critical point. During this period, the force decreases with increasing displacement, and the stiffness takes on a negative value until the deformation reaches another critical point. The V-shaped beams, which generate positive stiffness, are composed of two flexible straight beams connected at both ends. When the rigid stage moves, the flexible straight beam in the V-shaped beams only suffers a small torsional deformation and no tensile deformation.

As shown in Figure 1b, the upper and lower plate spring of bi-stable beams are parallel. There was no parallel relationship between the upper plate spring of the bi-stable beams and the lower plate spring of the V-shaped beams. Similarly, the other corresponding plate springs are not parallel to each other. In general, constructing a CFM required adjusting the geometrical parameters of the negative stiffness mechanism (bi-stable beam) and the positive stiffness mechanism (V-shaped beam) separately, including the shape parameters, in order to optimize the zero-stiffness performance. Therefore, $\theta_{w}\neq \theta_{v}/2$.

**Figure 2:**
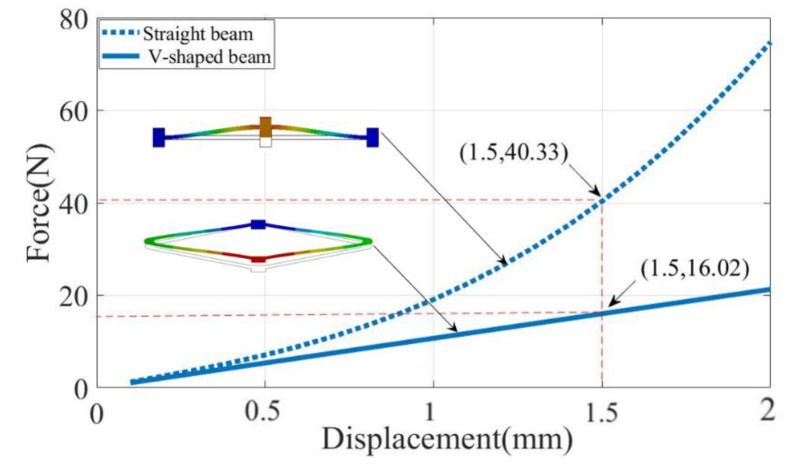
Comparison of the positive stiffness structure.

For the positive stiffness structure, we chose a flexible straight beam and a double V-shaped beam, and the comparative analysis was carried out by finite elements. As shown in Figure 2, it can be seen that the relationship between the force and displacement of the straight beam exhibits a significant nonlinear relationship. This is due to the fact that a certain amount of stress hardening occurs when a certain displacement is applied to a flexible straight beam. Therefore, the straight beam cannot be well matched with the negative stiffness part to obtain a stable output constant-force characteristic. The V-shaped beam structure exhibited higher linearity than the straight beam structure and could be better combined with the negative stiffness part to obtain constant-force characteristics. The use of double V-shaped beams also allowed for the avoidance of micro-slip due to non-integral coil spring assemblies, reducing parasitic motion around the perpendicular to the bi-stable beam, and could be designed to have linear stiffness characteristics with small deformations. At the same time, the output force of the straight beam was larger for the same dimensions. Meanwhile, in order to obtain an output force matching the bi-stable beam, the double V-shaped structure was chosen in this article.

### 2.2. Analytical Modeling of the CFM

To analyze the compliant CFM, it was necessary to establish mathematical models for the double V-shaped beam and the bi-stable beam, respectively, and to develop the stiffness model according to the principle of combining positive and negative stiffness. Then, an analytical model of the whole CFM could be established, and the relationship curve between the force and displacement could be obtained.

#### 2.2.1. V-Shaped Beam

**Figure 3:**
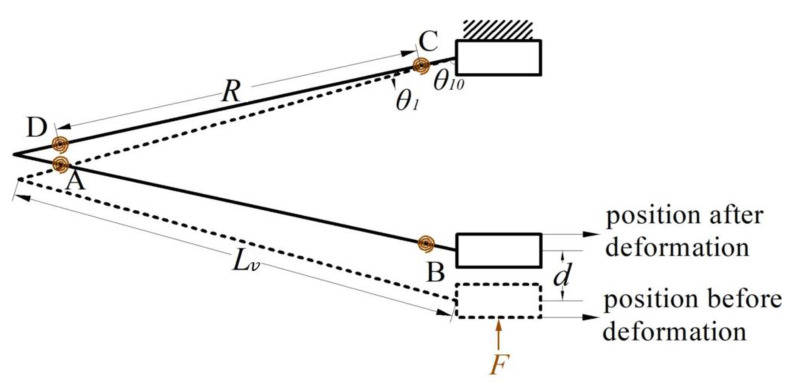
Pseudo-rigid body model of the V-shaped beam.

First, a pseudo-rigid body model (PRBM) was established for the double V-shaped beam [31]. Since the V-shaped beams are symmetrically distributed, one flexible beam was taken for structural analysis in order to simplify the modeling process, as shown in Figure 3. In the designed structure, the V-shaped beams are indirectly connected to the fixed end through the bi-stable beam mechanism. Therefore, in order to model and analyze the V-shaped beam, the end connected to the connecting block was fixed, and the other end was subjected to a force F. Meanwhile, we used the PRBM to analyze and build the stiffness model.

In this mechanism, the length of the two sides of the V-shaped beam is $L_{v}$; the torsion springs at the hinge points A–D reflect the deformation resistance of the beam; the flexible beam between the two torsion springs is the length R of the PRB rod, whose pseudo-rigid body angle is $\theta_{1}$; the initial angle between the end of the V-shaped beam and the vertical direction is $\theta_{10}$; the distance moved under the action of the force is d. On the basis of the PRB model shown in the figure, we conducted a theoretical analysis using the principle of virtual work, so as to model the stiffness of the double V-shaped beam.

As shown in Figure 3, the relationship between the PRB angle $\theta_{1}$ and the displacement d is as follows:

(1) $$d=2R\cos\theta_{10}-R\cos\left(\theta_{1}+\theta_{10}\right)$$

where $R=\gamma L_{v}\;$is the pseudo-rigid rod length and γ is the characteristic radius coefficient. Selecting $\theta_{1}$ as the generalized coordinate and differentiating the generalized coordinate from Equation (1), then the virtual displacement of the slider is as follows:

(2) $$\delta d=-2R\sin\left(\theta_{1}+\theta_{10}\right)\delta \theta_{1}$$

The virtual work performed by the slider under the action of the force F is as follows:

(3) $$\delta W_{F}=2FR\sin\left(\theta_{1}+\theta_{10}\right)\delta \theta_{1}$$

In the PRB model, the virtual work performed by the four torsion springs is as follows:

(4) $$\delta W_{T}=-4\kappa \theta_{1}2FR\delta \theta_{1}$$

Therefore, the total virtual work performed by the V-shaped beam is as follows:

(5) $$\delta W=\delta W_{F}+\delta W_{T}$$

Putting the above Equations (3) and (4) into Equation (5), we can obtain from the virtual work principle as follows:

(6) $$2FRsin\left(\theta_{1}+\theta_{10}\right)\delta \theta_{1}-4\kappa \theta_{1}\delta \theta_{1}=0$$

The relationship between the V-shaped beam force F and the PRB angle $\theta_{1}$ can be obtained by the following:

(7) $$F=\frac{2\kappa \theta_{1}}{R\sin\left(\theta_{1}+\theta_{10}\right)}$$

Then, the stiffness of the double V-shaped beam is as follows:

(8) $$k=\frac{F}{d}=\frac{2\kappa \theta_{1}}{R^{2}\sin\left(\theta_{1}+\theta_{10}\right)\left[\cos\theta_{10}-R\cos\left(\theta_{1}+\theta_{10}\right)\right]}$$

For the validity of Equations (7) and (8), we restrict the range of the relevant parameters (R, $\theta_{10}$, and $\theta_{1})$ of the V-shaped beam, respectively. The basic mechanism is designed to determine the constraints on the three parameters, referring to the design guidelines in the literature [18]. First, the length of the plate spring of the V-shaped beam is less than the bi-stable beam, i.e., $L_{v}$ < $L_{w}$. The value R is the pseudo-rigid length of the plate spring of the V-shaped beam, in general, $R=\gamma L_{v}$. Therefore, we can derive a range of constraints for R: 0 < R < $\gamma L_{w}$. Second, by the internal angle theorem of the isosceles triangle, we can derive a range of constraints for $\theta_{10}$: $0<\theta_{10}<90{}^{\circ}$. Finally, since in this paper, we input a displacement of 2.4 mm for the CFM, according to Equation (1) we can derive the restriction range for the rigid body angle $\theta_{1}$: $0<\theta_{1}<3{}^{\circ}$. Therefore, we restricted the range of values for the pseudo-rigid body length R, the pseudo-rigid body angle $\theta_{1}$, and the bottom angle $\theta_{10}$ of the V-shaped beam as follows:

(9) $$\left\{\begin{array}{c} 0<R<\gamma L_{w}\\ 0<\theta_{10}<90{}^{\circ}\\ 0<\theta_{1}<3{}^{\circ} \end{array}\right.$$

**Figure 4:**
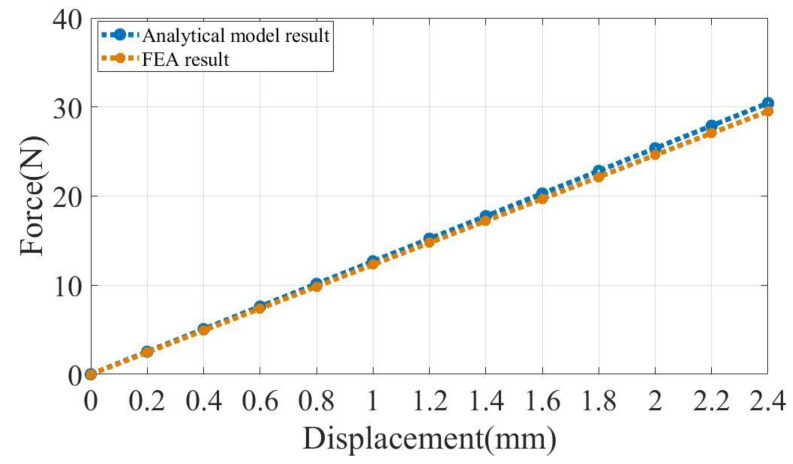
Relationship between the reaction force and displacement of the positive stiffness structure.

We conducted an analysis of the stiffness of the V-shaped beam by FEA simulation. In Figure 4, the relationship between force and displacement is demonstrated, and its slope represents the stiffness of the V-shaped beam. Within a certain range of input displacement, the force and displacement show a linear relationship, so its stiffness is constant.

#### 2.2.2. Bi-Stable Beam

The negative stiffness effect of a bi-stable beam is caused by the buckling characteristic, which is a nonlinear deformation. In this article, the bi-stable beams were theoretically modeled using the chained beam constraint model (CBCM) [32]. The flexible beam was discretized into several equal elements and modeled using a beam constraint model (BCM) for each beam element.

**Figure 5:**
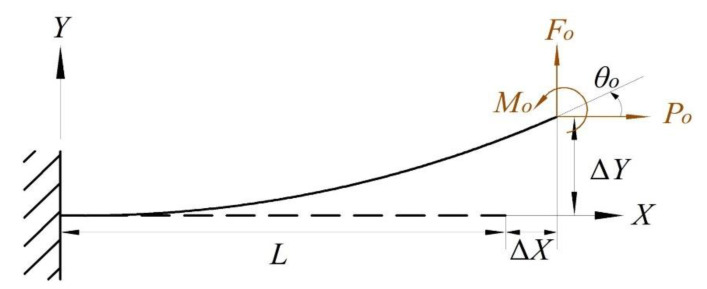
Typical beam with a planar flexible beam.

First, the CBCM was constructed based on the beam constraint model (BCM) proposed by Awtar [33], which is an effective solution for the non-linear deformation of flexible beams. According to the schematic diagram of a typical beam with a planar flexible beam shown in Figure 5, the CBM model is derived as follows:

(10) $$\left[\begin{array}{c} f_{o}\\ m_{o} \end{array}\right]=\left[\begin{array}{cc} 12 & -6\\ -6 & 4 \end{array}\right]\left[\begin{array}{c} \delta_{y}\\ \theta_{o} \end{array}\right]+p_{o}\left[\begin{array}{cc} 6/5 & -1/10\\ -1/10 & 2/15 \end{array}\right]\left[\begin{array}{c} \delta_{y}\\ \theta_{o} \end{array}\right]$$

(11) $$\delta_{x}=\frac{t^{2}p_{o}}{12L^{2}}+\frac{1}{2}\left[\begin{array}{cc} \delta_{y} & \theta_{o} \end{array}\right]\left[\begin{array}{cc} 3/5 & -1/20\\ -1/20 & 1/15 \end{array}\right]\left[\begin{array}{c} \delta_{y}\\ \theta_{o} \end{array}\right]+p_{o}\left[\begin{array}{cc} \delta_{y} & \theta_{o} \end{array}\right]\left[\begin{array}{cc} -1/700 & -1/1400\\ -1/1400 & -11/6300 \end{array}\right]\left[\begin{array}{c} \delta_{y}\\ \theta_{o} \end{array}\right]$$

To simplify the derivation of the control equations for the BCM, the load was normalized to the geometric and material parameters of the beam as follows:

(12) $$f_{o}=\frac{F_{o}L^{2}}{EI};\;p_{o}=\frac{P_{o}L^{2}}{EI};\;m_{o}=\frac{M_{o}L^{2}}{EI};\;\delta_{y}=\frac{\Delta Y}{L};\;\delta_{x}=\frac{\Delta X}{L}\;$$

**Figure 6:**
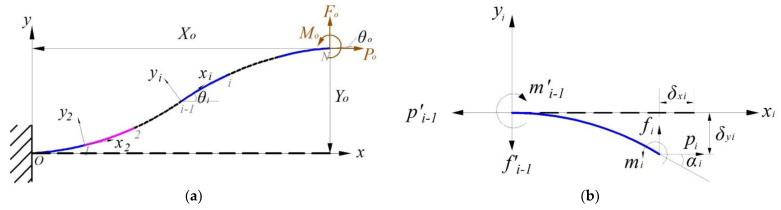
Discretization of the plane’s large deformation beam: (**a**) discretization analysis of the beam; (**b**) *i*th element.

As shown in Figure 6a, the end force and end bending moment of the flexible beam are denoted by $F_{o}$, $P_{o},$ and $M_{o}$. Respectively, the end displacement and end rotation angle of the flexible beam are denoted by $X_{o}$, $Y_{o},$ and $\theta_{o}$. Assuming that the plane’s flexible beam is divided into N beam elements of equal length, for the ith $\left(2\leq i\leq N\right)$ element, a local coordinate system $O_{i}x_{i}y_{i}$ is established at the end point (node i−1) of the (i−1)th element and along the tangent to the node i−1. The local coordinates of the first element were established at the fixed end of the plane flexible beam, that is, the node O, and the free end of the plane’s flexible beam is the node N. As shown in Figure 6b, we used $f_{i}$, $p_{i}$, and $m_{i}$ to represent the nodes in the local coordinate system of the ith segment’s normalized lateral force, normalized axial force, and normalized bending moment on the node i, respectively. Meanwhile, we used $\delta x_{i}$, $\delta y_{i}$, and $\alpha_{i}$ to represent the corresponding lateral displacement, axial displacement, and end rotation angle, respectively. The load balance equation for the ith column is as follows:

(13) $$\left[\begin{array}{c} f_{i-1}^{\prime }\\ p_{i-1}^{\prime }\\ m_{i-1}^{\prime } \end{array}\right]=\left[\begin{array}{ccc} 1 & 0 & 0\\ 0 & 1 & 0\\ 1+\delta_{xi} & -\delta_{yi} & 1 \end{array}\right]\left[\begin{array}{c} f_{i}\\ p_{i}\\ m_{i} \end{array}\right]$$

In this case, the transverse force, axial force, and bending moment of the (i−1)th unit on the ith unit are denoted by $f_{i-1}^{\prime }$, $p_{i-1}^{\prime }$, and $m_{i-1}^{\prime }$, respectively. Since the ith unit has a rigid body rotation $\alpha_{i-1}$ around the (i−1)th unit, we can obtain the following:

(14) $$\left[\begin{array}{c} f_{i-1}\\ p_{i-1}\\ m_{i-1} \end{array}\right]=\left[\begin{array}{ccc} \cos\alpha_{i-1} & -\sin\alpha_{i-1} & 0\\ \sin\alpha_{i-1} & \cos\alpha_{i-1} & 0\\ 0 & 0 & 1 \end{array}\right]\left[\begin{array}{c} f_{i-1}^{\prime }\\ p_{i-1}^{\prime }\\ m_{i-1}^{\prime } \end{array}\right]$$

The angle $\theta_{i}$($\theta_{1}$ = 0) that the ith element rotates around the global coordinate system can be expressed as follows:

(15) $$\theta_{i}=\overset{i-1}{\underset{1}{\sum }}\alpha_{i}$$

The simultaneous Equations (14) and (15) yield the equilibrium equation for the ith element (there are 3(N−1) equations in total):

(16) $$\theta_{i}=\overset{i-1}{\underset{1}{\sum }}\alpha_{i}\left[\begin{array}{ccc} \cos\theta_{i} & -\sin\theta_{i} & 0\\ \sin\theta_{i} & \cos\theta_{i} & 0\\ 1+\delta_{xi} & -\delta_{yi} & 1 \end{array}\right]\left[\begin{array}{c} f_{i}\\ p_{i}\\ m_{i} \end{array}\right]=\left[\begin{array}{c} f_{1}\\ p_{1}\\ m_{i-1} \end{array}\right]$$

Normalizing the end load (because the local coordinate system and the global coordinate system of the first element coincide, so the directions of $f_{1}$ and $p_{1}$ are parallel to the directions of $f_{o}$ and $p_{o}$, respectively), we obtain the following:

(17) $$f_{o}=\frac{F_{o}L^{2}}{EI}=N^{2}f_{1}\;p_{o}=\frac{P_{o}L^{2}}{EI}=N^{2}p_{1}\;m_{o}=\frac{M_{o}L^{2}}{EI}=Nm_{N}$$

The geometric constraint equations for the entire plane flexible beam can be written as follows (three equations in total):

(18) $$\left\{\begin{array}{c} \left\{\overset{N-1}{\underset{i=1}{\sum }}\left[\left[\begin{array}{cc} \cos\theta_{i} & -\sin\theta_{i}\\ \sin\theta_{i} & \cos\theta_{i} \end{array}\right]\left[\begin{array}{c} L_{i}\left(1+\delta x_{i}\right)\\ L_{i}\delta y_{i} \end{array}\right]\right]=\left[\begin{array}{c} X_{o}\\ Y_{o} \end{array}\right]\right.\\ \theta_{N}+\alpha_{N}=\theta_{o} \end{array}\right.$$

The length of the ith cell is $L_{i}$, and for equal-length divisions, there is $L_{i}=L/N$.

**Figure 7:**
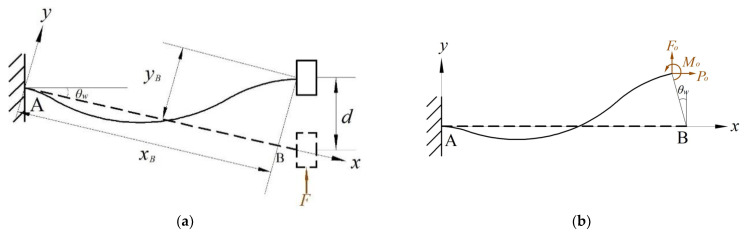
Schematic of the bi-stable beam: (**a**) schematic of the bi-stable beam; (**b**) force of the positive coordinate system.

As shown in Figure 7a, for this bi-stable beam, the global coordinate system was set at the fixed end A, with the *x*-axis along the beam length and the *y*-axis perpendicular to the beam. We divided the flexible beam into three sections and used the beam constraint model for modeling analysis. For a given guide end B, the lateral displacement is d, so the end displacement of the flexible beam can be expressed as follows:

(19) $$X_{o}=L_{AB}-d\sin\theta_{w}\;Y_{o}=-d\cos\theta_{w}\;\theta_{o}=0$$

As Figure 7b shows, the lateral force F at the leading end B can be expressed by the end loads ($F_{o}$ and $P_{o}$) of the beam as follows:

(20) $$F=F_{o}\cos\theta_{w}+P_{o}\sin\theta_{w}$$

**Figure 8:**
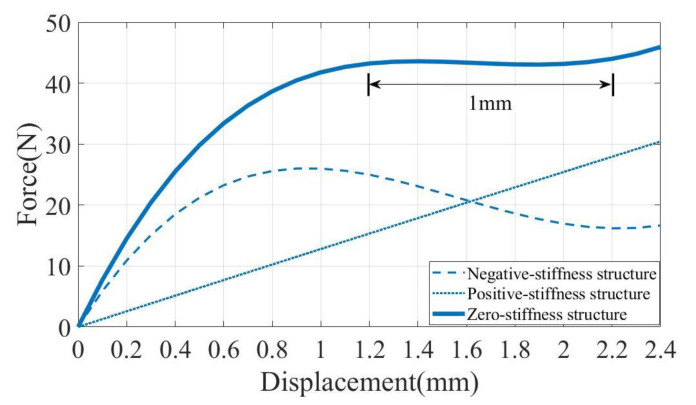
Analytical model results of the CFM.

The above theoretical analysis was carried out on the flexible bi-stable beam and the double V-shaped beam, and MATLAB software was used to draw the curve diagram. Finally, a curve with a constant-force range was formed, as shown in Figure 8.

## 3. FEA and Parameter Analysis

### 3.1. Finite Element Analysis

**Figure 9:**
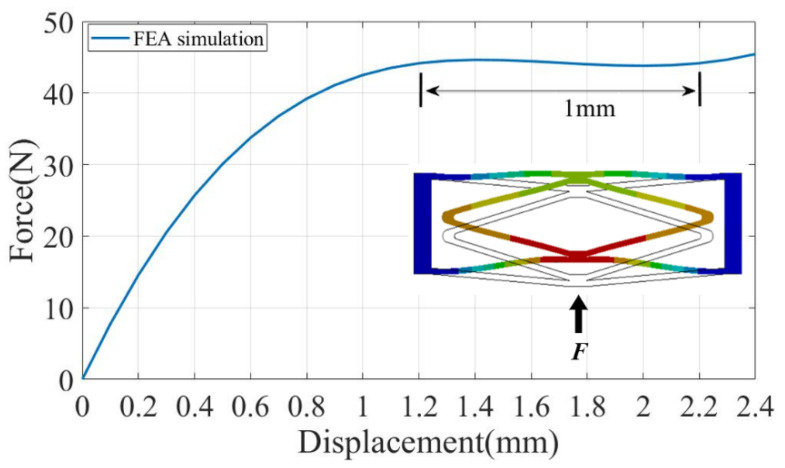
FEA results of the CFM.

In order to verify the performance of the compliant CFM, a nonlinear simulation study was conducted using the commercial software ANSYS. First, the model of the CFM was built. Then the model was imported into workbench and the material was set to aluminum alloy. The material parameters used for the aluminum alloy can be found in Table 1. A certain displacement was input at the lower end of the mechanism and the step size was set. The final reaction force was derived. The force–displacement curve of the CFM derived from the simulation is shown in Figure 9, which verifies that the CFM has certain characteristics of outputting constant force.

As shown in the force–displacement curve obtained through simulation, by applying a displacement of 2.4 mm at the bottom end of the CFM, the mechanism outputs a near-constant force of 44.2 N within 1 mm. The results of the FEA of the CFM match the analysis results of the mathematical model, which verifies the output constant-force characteristics of the designed CFM.

The geometric parameters of the CFM were determined by simulation. Additionally, the specific dimensional parameters are shown in Table 2.

### 3.2. Parameter Analysis

**Figure 10:**
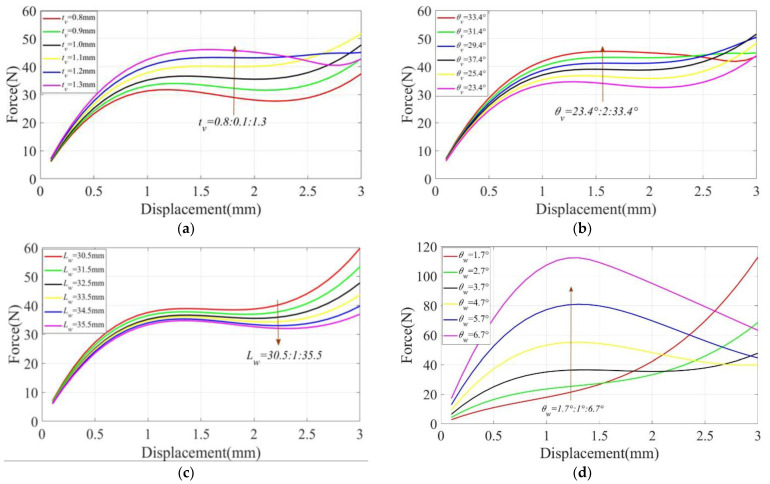
Institutional parameters: (**a**) $t_{v}$; (**b**) $\theta_{v}$; (**c**) $L_{w}$; (**d**) $\theta_{w}$.

The parameter of the structure significantly determined the output characteristics of the CFM [34]. In this subsection, we investigated the sensitivity of the parameter. It was clear from Equations (7) and (19) that the constant force characteristics were exceptionally sensitive to the parameters $t_{v}$, $\theta_{v}$, $L_{w}$, and $\theta_{w}.$ We changed the values of each of the four parameters. We changed the values of the four parameters and derived the output characteristic curves of the CFM using the finite element software ANSYS. As shown in Figure 10, we observed that the variation of different parameters had a determining effect on the output performance of CFM.

First, as shown in Figure 10a, $t_{v}$ increased from 0.8 mm to 1.3 mm with an interval of 0.1 mm. As $t_{v}$ increased, the force also increased and the relationship between $t_{v}$, and the force was approximately proportional. Within 0.8–1.1 mm, the change in $t_{v}$ had almost no effect on the constant-force strokes. Within 1.1–1.3 mm, the stiffness of the V-shaped beam increased due to the larger $t_{v}$. Therefore, the bi-stable beam on the upper side was pushed to move upward to reach the flexural state, so the curve changed, and the constant-force strokes became larger. Within 1.1–1.3 mm, the stiffness of the V-shaped beam increased due to the larger $t_{v}$. Therefore, the bi-stable beam on the upper side was pushed to move upward to reach the flexural state, so the curve changed, and the constant-force strokes became larger. Second, as shown in Figure 10b, $\theta_{v}$ increased from 23.4° to 33.4° at an interval of 2°. It can be seen that the effects of $\theta_{v}$ and $t_{v}$ on the CFM are the same, and the difference lies in the different effects on the magnitude of the force. $t_{v}$ had a more obvious effect on the magnitude of the force within the constant-force distinction, while the effect of $\theta_{v}$ on the magnitude of the force was relatively flat.

As shown in Figure 10c, the effect of the parameters of the bi-stable beam on the performance of the CFM was explored. First, the length $L_{w}$ of the bi-stable beam was spaced from 30.5 to 35.5 mm with an interval of 1 mm, increasing the constant-force strokes with the increase in $L_{w}$ and decreasing the magnitude of the force with the increase in the beam length. Second, as shown in Figure 10d, $\theta_{w}$ varied from 1.7° to 6.7° at 1° intervals, and it was observed that a larger $\theta_{w}$ increased the travel of the constant force. The magnitude and amplitude of the force also increased with the increasing $\theta_{w}$. This indicates that the magnitude of the inclination angle of the bi-stable beam has a greater effect on the size of the region generating negative stiffness.

## 4. Experimental Study

### 4.1. Prototype Fabrication

The designed CFM was machined by the wire-cutting process, and the processing material was Al-7075 aluminum alloy (Young’s modulus, 71 Gpa; Poisson’s ratio, 0.33). The magnitude of the constant force generated by selecting different materials will be different. Therefore, different materials can be selected according to the actual situation to achieve different constant forces for different occasions. Verification of the prototype was carried out on a pneumatic stage, where the device required for the experiment was mounted. The force sensor (SFS200) was used to measure the magnitude of the force and was bolted to the convex end connection block of the CFM, while the force sensor was bolted to the micro-positioning stage, and a certain displacement was given to the CFM by turning the X–Y positioning stage. The displacement was measured by a digital indicator meter (Mitutoyo 543-390B). The force sensor was connected to the computer terminal through a high-frequency data collector (LabPro508) to monitor the magnitude of the generated force in real-time to verify the performance of the designed CFM.

**Figure 11:**
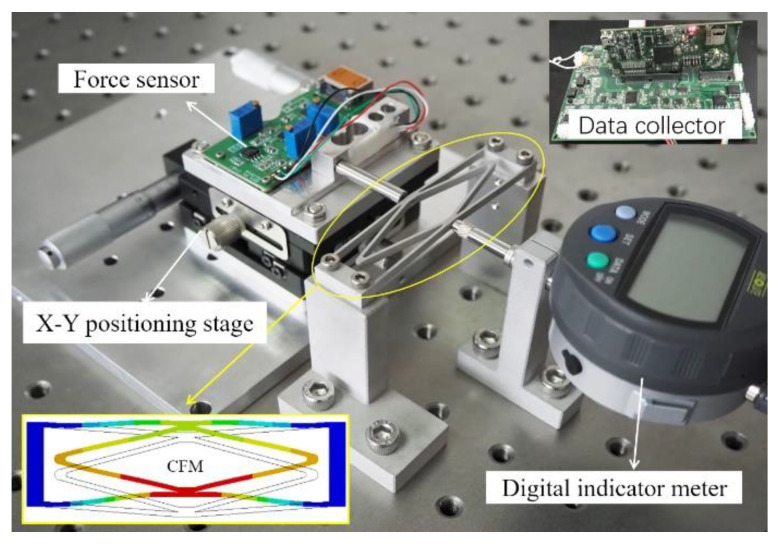
The experimental apparatus.

**Figure 12:**
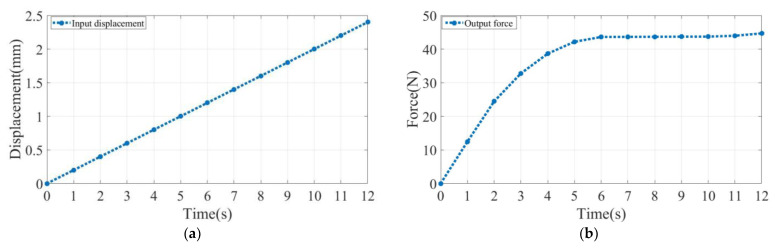

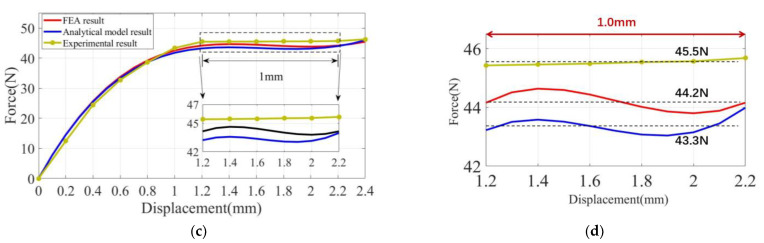
Experimental results: (**a**) displacement output; (**b**) force output; (**c**) constant-force properties of zero-stiffness structures; (**d**) enlarged view of the constant force zone.

As shown in Figure 11, we moved the force sensor through the positioning stage, and the experiment started when the force sensor contacted the bottom of the CFM. After the force sensor contacted the CFM, we continued to move the positioning stage for the same time and recorded the data. At this point, the CFM generates a reaction force on the force sensor, which is processed by the data collector and reflected on the display. In the process, we recorded the input displacement and output reaction force for each time period. Additionally, it was selected 13 points in the same time interval to plot the curves of input displacement and output force in relation to time (Figure 12a,b). Finally, the two curves were fitted to the desired constant force characteristic curve (Figure 12c).

### 4.2. Experimental Results

In this section, the CFM was experimentally tested, and its performance was discussed. A displacement of 2.4 mm was input to the end of the CFM, as shown in Figure 12a. The output constant force measured by the force sensor was 45.5 N, as shown in Figure 12b, and the constant force ranged between 1.2 and 2.2 mm. It can be seen from Figure 12c that the CFM designed in this paper has the characteristic of outputting constant force for a period of time. The experimental results agree closely with the simulation and theoretical results, and the performance of the structure was verified. In Figure 12d, we enlarged the view of experimental results to clearly demonstrate the constant force zone. Due to the manufacturing error causing the experimental results to have a certain error against the simulation theory, the manufacturing error, material properties, and surface roughness, and other imperfections are the main reasons for the deviation between the model and experimental results.

In Table 3, we present a comparison of the variability between the model, theory, and simulation, respectively, and quantify the error of the three results in the constant force zone. Compared with the experimental results, there existed a negligible error of less than 5% for the constant force derived from the theoretical and simulated results. It can conclude that the designed mechanism has a certain performance of the outputting constant force.

## 5. Conclusions

In this paper, a novel nested, compliant CFM with a millimeter-level stroke was proposed, which can overcome the limitations of the small output force and constant-force strokes of the existing CFM. The configuration uses the principle of combining positive and negative stiffnesses to generate a constant output force, and the positive stiffness mechanism is located within the negative stiffness mechanisms. We analyzed the mechanism in detail. The positive and negative stiffness parts of the CFM were modeled and analyzed using the method of PRB and CBCM, respectively. A simulation analysis of the static mechanics was also carried out using finite element software. Finally, a prototype was fabricated, and an experimental platform was built for performance testing. The experimental results showed that the constant-force mechanism has constant-force strokes of the millimeter level and can output a constant force of 45.5 N within 1 mm of a stroke.

In Table 4, we compare the literature on the related constant-force mechanism, and the materials used for the prototype fabrication included aluminum alloy and 3D printing materials. As can be seen from the table, the CFMs covered in the literature all have relatively small output forces and different output stroke lengths. The CFM designed in this article is capable of outputting a constant force of 45.5 N within millimeter strokes, which can meet the needs of operating hard-surface materials. In future research, we will optimize the mechanism to obtain larger constant-force strokes to suit more occasions.

## Figures and Tables

**Table 1:** Table of the parameters of the aluminum alloy.

Parameters	Numerical Value
Young’s modulus	71 Gpa
Density	2810 kg/m^3^
Poisson’s ratio	0.33

**Table 2:** Table of the parameters of the CFM.

Parameters		Numerical Value
Bi-stable beam	Length $L_{w}$	32.5 mm
	Tilt angle $\theta_{w}$	3.7°
	Thickness $t_{w}$	1.0 mm
	Width ω	3.1 mm
V-shaped beam	Length $L_{v}$	27.22 mm
	Angle $\theta_{v}$	25.4°
	Thickness $t_{v}$	1.1 mm
	Width ω	3.1 mm

**Table 3:** Analysis of results in the constant force interval.

Performance	Experiment	FEA	Model	Error
Constant force (N)	45.5	44.2	-	5%
		-	43.3	3%
Constant stroke (mm)	1.0	1.0	1.0	-

**Table 4:** Comparison of the existing literature.

Designs	Constant Force (N)	Stroke (mm)
This work	45.5	1.0
Literature [27]	29	0.7
Literature [18]	11.92	2.13
Literature [35]	11.15	2
Literature [22]	0.53	0.22
Literature [36]	0.3	0.65

## Data Availability

The data presented in this study are available on request from the corresponding author. Due to specific data of the experiment have been published in the article.
